# Arthroscopic labral debridement versus labral repair for patients with femoroacetabular impingement: A meta-analysis

**DOI:** 10.1097/MD.0000000000020141

**Published:** 2020-05-08

**Authors:** Zhan-Xiong Wu, Wen-Xia Ren, Yi-Ming Ren, Meng-Qiang Tian

**Affiliations:** a Department of Orthopedics, Shanxi Bethune Hospital & Shanxi Academy of Medical Sciences; b Endocrine and Metabolic Center, Taiyuan Central Hospital, Taiyuan, Shanxi Province; c Department of Joint and Sport Medicine, Tianjin Union Medical Center, Tianjin, P.R. China.

**Keywords:** acetabular labrum, femoroacetabular impingement, hip arthroscopic surgery, meta-analysis, systematic review

## Abstract

**Objective:**

Femoroacetabular impingement (FAI) is a common cause of hip pain and even tearing of the acetabular labrum in young adults and athletes. Either arthroscopic labral debridement (LD) or labral repair (LR) technique for FAI patients is needed to choose. We conducted this systematic review and meta-analysis to compare the clinical outcomes of arthroscopic LD versus LR intervention.

**Methods:**

The five studies were acquired from PubMed, Medline, Embase, and Cochrane Library. The data were extracted by two of the coauthors independently and were analyzed by RevMan5.3. Mean differences (MDs), odds ratios (ORs), and 95% confidence intervals (CIs) were calculated. Cochrane Collaboration's Risk of Bias Tool and Newcastle–Ottawa Scale were used to assess risk of bias.

**Results:**

Four observational studies and one prospective randomized study were assessed. The methodological quality of the trials indicated a low to moderate risk of bias. The pooled results of Non-Arthritic Hip Score (NAHS), failure rate of surgeries and complications showed that the differences were not statistically significant between the two interventions. The difference of modified Harris Hip Score (mHHS), the Visual Analogue Scale (VAS) score and satisfaction rate was statistically significant between LD and LR intervention, and LR treatment was more effective. Sensitivity analysis proved the stability of the pooled results and there were too less included articles to verify the publication bias.

**Conclusions:**

Hip arthroscopy with either LR or LD is an effective treatment for symptomatic FAI. The difference of mHHS, VAS score, and satisfaction rate was statistically significant between LD and LR intervention, and arthroscopic LR could re-create suction-seal effect, potentially reduce microinstability, which demonstrated a trend toward better clinical efficacy and comparable safety compared with LD. The arthroscopic LR technique is recommended as the optical choice for acetabular labrum tear with FAI.

## 1 Introduction

In the late 1990s, Ganz and Beck et al^[1,2]^ mentioned the concept of femoroacetabular impingement (FAI), which is a mechanical process that occurs due to anatomical variation at the acetabulum and the femoral head–neck junction. FAI is the common cause of hip pain for young people, especially athletes. According to the mechanism of injury, FAI can be divided into three types: cam type, pincer type, and mixed type.^[3,4]^ The acetabular labrum is an important structure that deepens the acetabular socket and ensures adequate load distribution within the hip joint. Importantly, it is easy to cause the damage of articular cartilage and tearing of the acetabular labrum after repetitive impact, eventually leading to osteoarthritis.^[5–9]^

Surgical treatment of labral damage due to FAI can be either labral debridement (LD) or labral repair (LR), and is still controversial. Up to now, some clinical studies compared radiographic and functional outcomes between arthroscopic LD and LR techniques. Some reported revealed that short to midterm results of LD in nonarthritic hips showed functional superiority compared with LR.^[10–12]^ In contrast, Espinosa et al^[13]^ hold that patients treated with labral refixation/repair recovered earlier and had superior clinical and radiographie results when compared with patients who had undergone resection of a torn labrum. However, there have been no systematic, quantitative evaluations between two techniques. In this article, we included five relevant studies to compare the clinical outcomes of arthroscopic LD and LR techniques in FAI to provide some evidence for clinical decision making.

## 2 Materials and methods

Ethical approval or patient consent was not required since the present study was a review of previous published literatures.

### 2.1 Inclusive criteria of published studies

#### 2.1.1 Types of studies

We considered all published and unpublished studies covering randomized controlled trials (RCTs), and observational studies including retrospective and prospective studies.

#### 2.1.2 Types of participants

All patients had been diagnosed as FAI on the basis of history, positive impingement signs on examination, and radiographic evidence and the presence of labral tear/pathology was required on magnetic resonance imaging (MRI), regardless of the etiology of the disease, associated pathology, gender, and age.

#### 2.1.3 Types of interventions

All surgical techniques including the arthroscopic labral resection/debridement technique and the arthroscopic labral refixation/repair/reconstruction technique were considered. The exclusion criteria were as follows:

1. insufficient clinical outcome data in studies and
2. reviews, letters or conference articles.

#### 2.1.4 Types of outcome measures

The primary outcome measures were the clinical outcomes synthesizing the Non-Arthritic Hip Score (NAHS), the modified Harris Hip Score (mHHS), the Visual Analogue Scale (VAS) score, and satisfaction rate. The secondary outcomes included: failure rate of surgeries and complications.

### 2.2 Search methods for identification of studies

Four databases (PubMed, Medline, Embase, and Cochrane Library) were searched using the keywords such as “ FAI or impingement syndromes or Femoro-Acetabular,” “labral resection or debridement,” “labral refixation or repair or reconstruction,” “surgery or surgical or operation,” and “arthroscopic or arthroscopy” through April 2019 to collect relevant studies about the clinical comparisons of LD versus LR intervention in FAI. The titles and abstracts of potential related articles identified by the electronic search were reviewed. References from retrieved articles were also assessed to extend the search strategy.

### 2.3 Data collection and quality assessment

Two partners (ZXW and YMR) independently assessed the titles and abstracts of all the studies screened during initial search, and they excluded any clearly irrelevant studies using the inclusion criteria. Data were independently extracted using a standard data form for the first author's name, year of publication, sample size, gender, age, intervention, country, study design, follow-up, and relevant outcomes. A third partner (MQT) would handle any disagreement about inclusion of a study and reach a consensus. Cochrane Collaboration's Risk of Bias Tool was manipulated for the appraisal of RCT study quality. Observational studies were assessed by the Newcastle–Ottawa Scale including 8 items. 12 A higher overall score indicates a lower risk of bias and a score of 5 or less (out of 9) corresponds to a high risk of bias.

### 2.4 Statistical analysis

RevMan statistical software 5.3 was used for meta-analysis. The continuous variables would be conducted by mean difference (MD) and 95% confidence interval (CI). For the dichotomous outcome, we calculated the odds ratios (ORs) and 95% CIs. The chi-squared statistic and the *I*^2^ statistic were used for the test of heterogeneity. A *P* < .05, *I*^2^ > 50% was considered a significant heterogeneity, and random-effect models were applied. Otherwise fixed-effect models were used if there was no significant heterogeneity (*P* ≥ .05, *I*^2^ ≤ 50%). We also performed sensitivity analysis by omitting one study at a time to test the stability of the pooled results. Publication bias was showed by the funnel plot.

## 3 Results

### 3.1 Studies identification and inclusion

**Figure 1:**
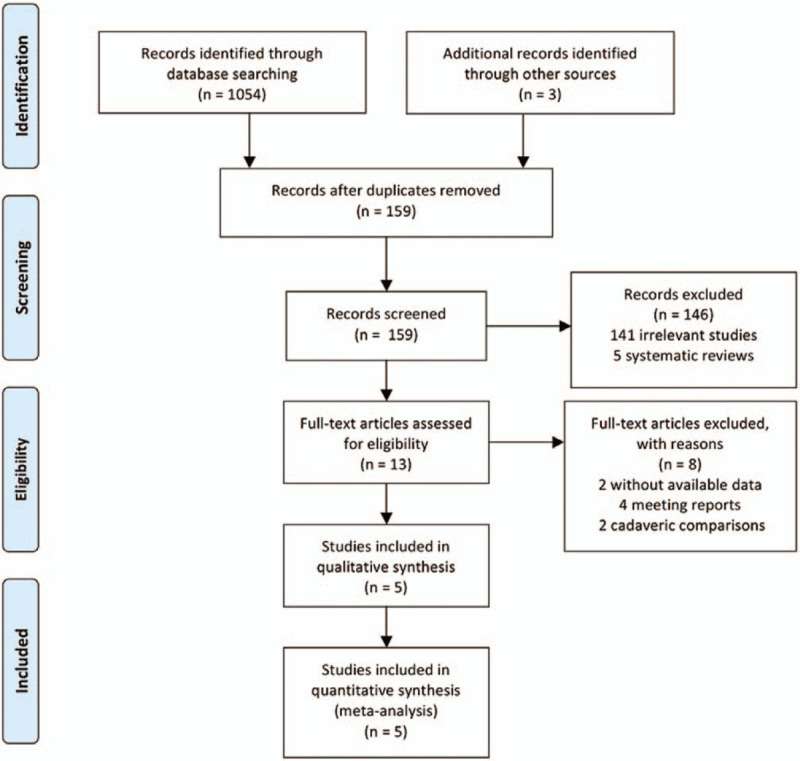
PRISMA flow diagram.

Searches conducted in the PubMed, Medline, Embase, Cochrane Library databases, and other sources, yielded a total of 1057 articles. After removing duplicates, 159 literatures were remained. Based on the titles and abstracts review, 141 irrelevant articles and 5 systematic reviews of them were excluded. Thirteen full-text articles were assessed for eligibility. However, eight articles were excluded based on the previously established exclusion criteria (2 without available data, 4 meeting reports and 2 cadaveric comparisons). Finally, five trials (1 RCT and 4 observational studies) were included in this systematic review and meta-analysis. The detail of selection process is listed in Figure 1.

### 3.2 Study characteristics

We assessed 5 studies^[14–18]^ including 1 RCT and 4 retrospective studies in this article. The included studies were conducted in 3 countries (Turkey, USA, and France) from 2009 to 2015, and involved 323 patients (171 patients treated with LD technique, 152 patients treated with LR technique) aged 28 to 39.5 years. The average follow-up duration ranged from 26.4 to 58.3 months. The clinical outcomes of the studies were evaluated mainly based on NAHS, mHHS, VAS score, satisfaction rate, and complications. The detailed information of included studies is shown in Table 1. In addition, Tonnis grade, Outerbrideg grade, and Coleman Score of our included were also assessed in Table 2.

**Table 1:**
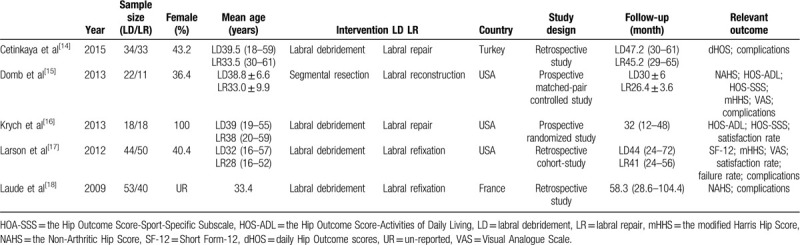
Characteristics of studies included.

**Table 2:**
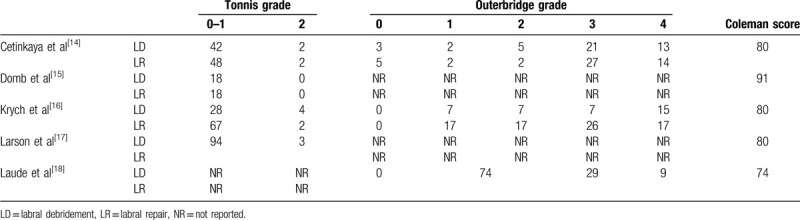
Basic information of included cases.

### 3.3 Methodological assessment of study quality

**Figure 2:**
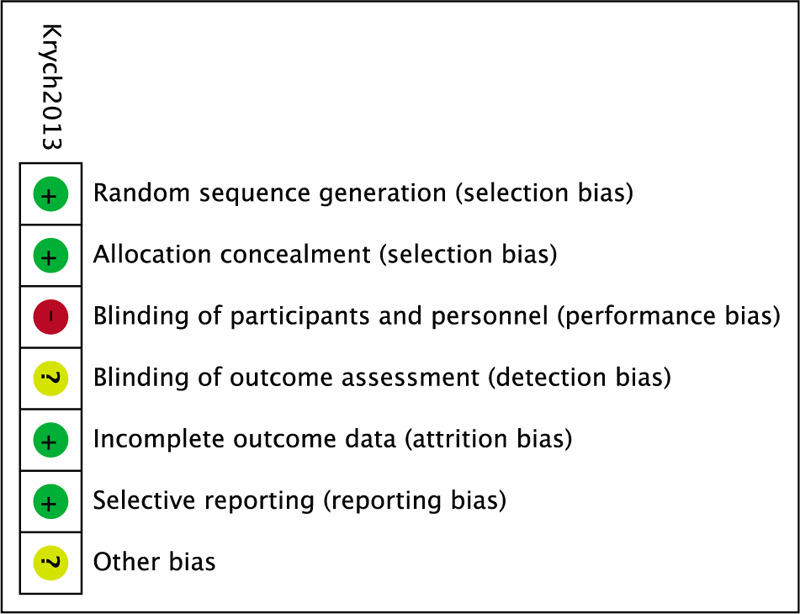
Risk of bias summary: this risk of bias tool incorporates the assessment of randomization (sequence generation and allocation concealment), blinding (participants and outcome assessors), incomplete outcome data, selective outcome reporting, and other risk of bias. The items were judged as “low risk,” “unclear risk,” or “high risk.” Green means “low risk,” red means “high risk,” and yellow means “unclear risk.”

Methodological quality assessment of the five included studies is presented in Figure 2 and Table 3. Among the RCT, Krych's study^[16]^ clearly described randomization was carried out opening one of 36 sealed, opaque envelopes assigning by patients to receive either LR or debridement, but the surgeon and patient were not blinded, which could be regarded as a moderate quality study. Among the observational studies, the Newcastle–Ottawa Scale including the exposed cohort, the non-exposed cohort, ascertainment of exposure, outcome of interest, comparability, assessment of outcome, length of follow-up, and adequacy of follow-up, was used to assess the risk of bias. The scores of all 4 studies ranged from 8 to 9, indicating a low risk of bias.

**Table 3:**
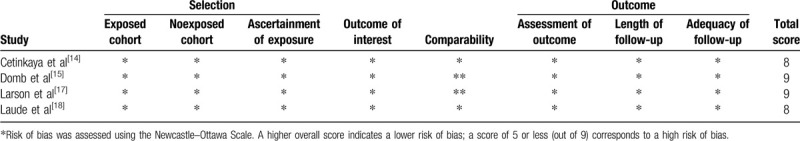
Risk of bias was assessed using the Newcastle–Ottawa Scale.

### 3.4 Comparison of NAHS between LD and LR

**Figure 3:**
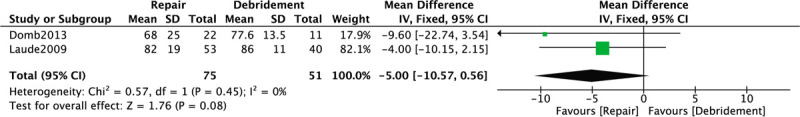
Forest plot of comparison: the Non-Arthritic Hip Score (NAHS) between arthroscopic labral debridement (LD) and labral repair (LR) technique.

Comparison of postoperative NAHS between LD and LR was conducted among the two included studies,^[15,18]^ which included 126 patients (75 patients receiving LD and 51 patients receiving LR), as shown in Figure 3. Heterogeneity testing showed that there was no heterogeneity among the studies (*P* = .45, *I*^2^ = 0%), so the fixed-effect model was used to pool the data from the 2 studies. The pooled result showed that the difference was not statistically significant between the LD group and the LR group (MD = −5.0, 95% CI = −10.57–0.56, *P* = .08).

### 3.5 Comparison of mHHS between LD and LR

**Figure 4:**
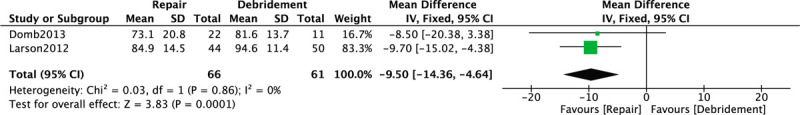
Forest plot of comparison: the modified Harris Hip Score (mHHS) between arthroscopic labral debridement (LD) and labral repair (LR) technique.

Comparison of postoperative mHHS between LD and LR was conducted between the two included studies,^[15,18]^ which enrolled 127 patients (66 patients receiving LD and 61 patients receiving LR), as shown in Figure 4. Heterogeneity testing showed that there was no heterogeneity between the studies (*P* = .86, *I*^2^ = 0%), so the fixed-effect model was used to pool the data for the two groups. The overall estimate showed that the difference was statistically significant between the LD group and the LR group (MD = −9.5, 95% CI = −14.36 to −4.64, *P* = .0001), and LR group had better results.

### 3.6 Comparison of VAS score between LD and LR

**Figure 5:**
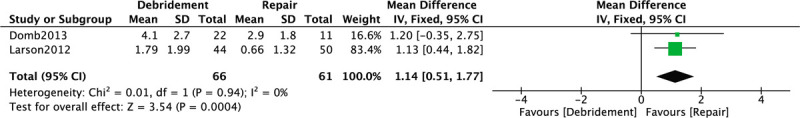
Forest plot of comparison: the Visual Analogue Scale (VAS) score between arthroscopic labral debridement (LD) and labral repair (LR) technique.

Comparison of postoperative VAS score between LD and LR treatment was conducted among two included studies^[15,16]^ which contain 127 patients in Figure 5. A heterogeneity test showed that there was no heterogeneity among studies (*P* = .94, *I*^2^ = 0%), so the random-effect model was used. The overall estimate showed that the difference between the two groups was statistically significant (MD = 1.14, 95%CI = −0.51–1.77, *P* = .0004).

### 3.7 Comparison of satisfaction rate between LD and LR

**Figure 6:**
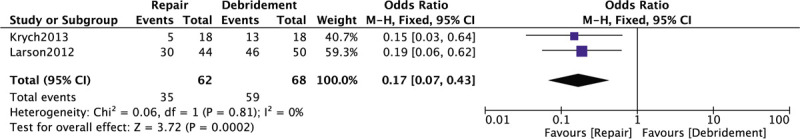
Forest plot of comparison: the satisfaction rate between arthroscopic labral debridement (LD) and labral repair (LR) technique.

In Figure 6, two included studies^[16,17]^ consisting of 127 patients (66 patients received LD treatment and 61 patients received LR treatment) investigated postoperative satisfaction rate. None heterogeneity among studies (*P* = .81, *I*^2^ = 0%) was found, so we used the fixed-effect model to pool the data. The overall estimate indicated that the pooled OR was 0.17 (95%CI = 0.07–0.43, *P* = .0002), suggesting that LD and LR treatment had a statistically significant difference, and LR performed better.

### 3.8 Comparison of failure rate of surgeries between LD and LR

**Figure 7:**
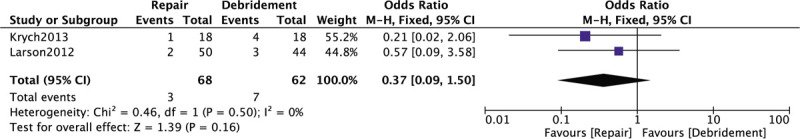
Forest plot of comparison: the failure rate of surgeries between arthroscopic labral debridement (LD) and labral repair (LR) technique.

Comparison of failure rate of surgeries between LD and LR was conducted among the two included studies,^[16,17]^ which included 132 patients (62 patients receiving LD and 70 patients receiving LR), as shown in Figure 7. Heterogeneity testing showed that there was no heterogeneity among the studies (*P* = .50, *I*^2^ = 0%), so the fixed-effect model was used to pool the data from the 2 studies. The pooled result showed that the difference was not statistically significant between the LD group and the LR group (OR = 0.37, 95% CI = 0.09–1.50, *P* = .16).

### 3.9 Comparison of complications between LD and LR

**Figure 8:**
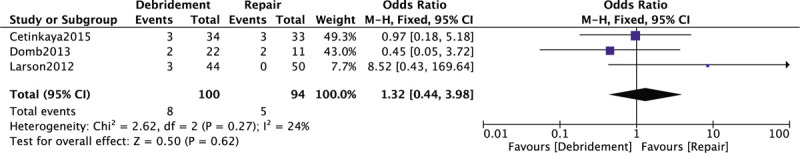
Forest plot of comparison: the postoperative complications between arthroscopic labral debridement (LD) and labral repair (LR) technique.

In Figure 8, three included studies^[14,15,17]^ consisting of 194 FAI patients (100 patients received LD and 94 patients received LR technique) reported complications. Low heterogeneity among studies (*P* = .27, *I*^2^ = 24%) was found, so we used the fixed-effect model. The overall estimate indicated that the pooled OR was 1.32 (95%CI = 0.44–3.98, *P* = .62), suggesting that the difference was not statistically significant.

### 3.10 Sensitivity analysis and publication bias

**Figure 9:**
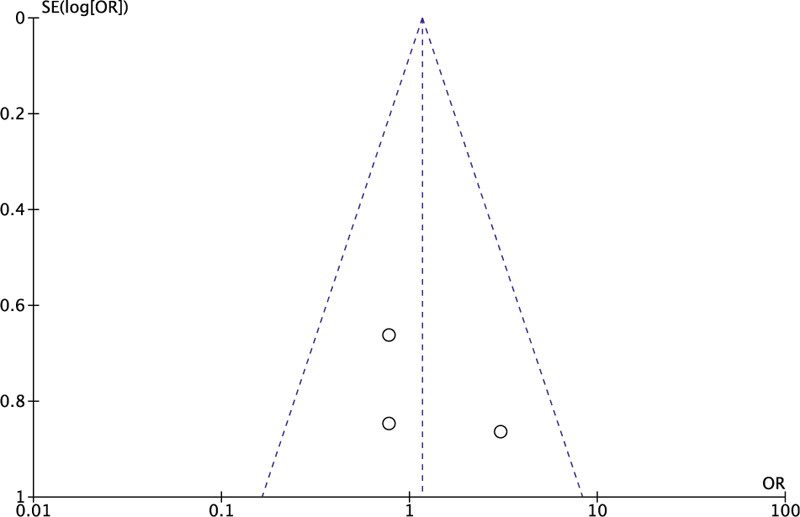
Funnel plot to test for publication bias. Each point represents a separate study for the indicated association. The vertical line represents the mean effects size. OR = odds ratio, SE = standard error.

We performed a sensitivity analysis to assess the stability of the pooled results. Among the most studies, the heterogeneity results were not obviously altered after sequentially omitting each study, indicating that our results were statistically reliable. The funnel plot of the included studies is shown in Figure 9. The points in the funnel plot were almost symmetrically distributed. However, too less included articles lead to an unbelievable result, and the publication bias could not be ignored.

## 4 Discussion

### 4.1 Summary of main results

Labral tear is generally secondary to FAI, trauma, dysplasia, capsular laxity, and degeneration. Patients with labral tear complain about anterior hip or groin pain most commonly. Conservative treatment consists of rest, non-steroidal anti-inflammatory medication, pain medications, modifications of activities, physical therapy, and intra-articular injection. When fail to respond to conservative treatment, surgical treatment including LD and LR is often indicated.^[19]^ In this study, we identified 1 RCT and 4 observational studies for investigating the clinical outcomes of arthroscopic LD versus LR intervention. Our meta-analysis results showed that the differences were not statistically significant between the two interventions for NAHS. However, a different result was discovered by mHHS, VAS score and satisfaction rate analysis. The difference of mHHS, VAS score, and satisfaction rate was statistically significant between LD and LR intervention, and the LR technique proved it had a higher efficacy. Labral debridement/focal resection is technically uncomplicated, but does not re-create suction-seal effect, and may result in compromised load distribution, shear stress. However, Labral reconstruction/repair could re-create suction-seal effect, potentially reduce microinstability, and show trend to superior clinical outcome.^[20]^

Although LD/segmental resection may successfully lead to symptom resolution, biomechanical testing supports the importance of labral preservation or reconstruction to optimize and normalize arthro kinematics. Distraction testing has shown that labral reconstruction and repair are superior to segmental resection,^[21]^ whereas axial compression tests are controversial. With compression and external rotation, LR has shown superiority to reconstruction and resection.^[22]^ However, in contrast, measurement of intra-articular fluid pressurization on compression suggests that reconstruction is superior to LR.^[23]^ Although labral preservation has been shown to produce durable results and, at this time, seems to be preferable to debridement/segmental resection, certain clinical circumstances may preclude labral preservation. In cases in which the acetabular labrum is unsalvageable, labral reconstruction/repair is supported clinically and biomechanically as an option to optimize joint mechanics, improve function, and promote pain mitigation for select individuals. Herickhoff et al^[24]^ found the intraoperative appearance of the labrum is the most important factor affecting surgical decision making. However, different surgeons viewing the same tear arthroscopically may select different treatments. The indications to repair a torn acetabular labrum are highly variable among hip arthroscopic surgeons. With regard to irreparable labral lesions, debridement of the damaged labrum is obviously not a preferable option while the LR associated with significant improvement in young patients and athletic populations.^[25,26]^ Long-term follow-up results with higher quality studies will be necessary to further define the role of labral reconstruction in hip preservation surgery.

The complications in five included studies also should be discussed. On the whole, 8 (8%) complications under LD surgery was reported and 5 (5.3%) complications under LR surgery was reported in 3 included studies.^[14,15,17]^ In Cetinkaya's study,^[14]^ 6 patients developed transient nerve palsy; 2 femoral nerve palsy cases of which 1 required surgical release, 2 pudendal nerve cases and 2 obturator nerve palsy cases at an average of 3 months of follow-up. In Domb's study,^[17]^ two patients in the arthroscopic labral reconstruction group had medial knee pain at the graft harvest site that resolved at 6 weeks’ follow-up. Two patients in the arthroscopic segmental labral resection group had superficial wound infections that were treated successfully with oral antibiotics. In Larson's study,^[17]^ 3 patients in the debridement group who developed heterotopic bone postoperatively. In addition, the failure rate or revision rate was 9.1% (4 hips) in the debridement group compared with 8.0% (4 hips) in the refixation group among Larson's study.^[17]^ Two patients subsequently had revision hip arthroscopy and two other patients in the debridement group underwent revision femoral osteochondroplasty. In the repair group, one patient underwent total hip arthroplasty and one patient subsequently underwent revision hip surgery. One patient in the LD group and one patient in the labral fixation group had undergone revision in Cetinkaya's study.^[14]^ One patient in the arthroscopic labral reconstruction group and two patients in the arthroscopic segmental labral resection group underwent revision arthroscopic surgery in Domb's study.^[15]^ In addition, our results talked about comparison of failure rate of surgeries between LD and LR, and none difference was found between them. In terms of the difficulty of operation, the difficulty of LR is higher than that of LD. The failure rate of LR operation is higher than that of LD group due to the limitation of the operator's personal level. Anatomically, the arthroscopic LR is closer to recovery normal acetabular labium structure, which contributes to the stability of the hip joint, the coordination of the movement and the secretion of the joint fluid. The probability of the recurrence of the hip joint pain and the failure rate is small after LR than LD.^[27–29]^ Interestingly, Menge et al^[30]^ reported higher rates of conversion to THA were seen in older patients, patients treated with acetabular microfracture, and hips with ≤2 mm of joint space preoperatively, regardless of labral treatment.

### 4.2 Limitations of the study

Some limitations of this study should be noted. First, the small sample size might have affected the significant difference between the two surgical procedures. Second, the included retrospective design and the patient cohorts were not similar because one group had a fixable labrum and the other did not, which may increase the clinical heterogeneity among trials. Third, our study only included 3 articles for conducting funnel plot and the publication bias could not be ignored. Last but not least, the included studies were mostly observational studies and not RCTs, and they largely relied on retrospectively collected data, resulting in a high risk of selection bias. More large-sample, multi-center, high-quality, RCTs are needed to verify the outcomes of this meta-analysis.

## 5 Conclusions

In conclusion, arthroscopic LD and LR are viable options for the treatment of acetabular labrum tear with FAI. LR treatment is more effective and have comparable complications compared with LD treatment, which indicates that the arthroscopic LR technique could be recommended as the optical choice for FAI. However, larger studies with longer follow-up are needed.

## Author contributions

YMR and ZXW conceived the design of the study. ZXW and YMR performed and collected the data and contributed to the design of the study. ZXW and YMR analyzed the data. YMR, WXR and MQT prepared and revised the manuscript. All authors read and approved the final content of the manuscript.
